# Design for extreme scalability: A wordless, globally scalable COVID-19 prevention animation for rapid public health communication

**DOI:** 10.7189/jogh.10.010343

**Published:** 2020-06

**Authors:** Maya Adam, Till Bärnighausen, Shannon A McMahon

**Affiliations:** 1 Stanford School of Medicine, Department of Pediatrics, Stanford University, Stanford, California, USA; 2 Institute of Global Health, Heidelberg University, Heidelberg, Germany; 3 Harvard T.H. Chan School of Public Health, Department of Global Health and Population, Harvard University, Boston, Massachusetts, USA; 4 Africa Health Research Institute, Wellcome Trust, Durban, South Africa; 5 Bloomberg School of Public Health, Johns Hopkins University, Baltimore, Maryland, USA

To scale globally, public health communication interventions must capture the public’s attention, meet them where they consume information and convey reliable messages in ways that are accessible across languages, ages, cultural affiliations and education levels. Novel approaches are needed to ensure that such interventions are developed and deployed quickly, sidestepping the typical delays associated with sluggish public health campaigns. Delays in producing compelling, evidence-based health communication can leave a vacuum that is quickly filled with misinformation. In the case of COVID-19, we watched the social media stratosphere explode with deceptive, anecdotal health messages that played on paranoia, fear and stigmatization, further aggravating a global state of unease and uncertainty. Social media personalities suggested anecdotal preventive strategies, ranging from the ineffective to the life-threatening.

For evidence-based recommendations to be heard above the anecdotal noise, they must be equal parts compelling and understandable to diverse global viewers. When health messages are packaged didactically, relying predominantly on the technical presentation of information, we run the risk of missing the audiences that need these messages the most. Framing health messages as informational arguments has proven to be less effective for changing behaviors than, for example, packaging recommendations within a relatable story [1,2]. Even the use of spoken or written text creates barriers, necessitates translation and slows the speed at which evidence-based health messages can be spread across social media networks.

By leaning on best practices from entertainment-education, communication theory [3,4], and even the commercial animation industry [5] health educators and health communication professionals can rapidly create compelling, evidence-based health messages that virtually “go viral”, while preventing their target diseases from doing the same.

In this commentary, we describe our experiences developing a wordless, globally scalable COVID-19 prevention animation on behalf of Stanford Medicine. We outline our process and experiences in hopes that it inspires fellow health communicators to authentically prioritize the needs of the broadest possible target audience. Our video, launched on 22 March 2020, was viewed and shared more than 1.2 million times across worldwide social media platforms within 10-day of its release. We emphasize three points in this commentary.

First, rapid design of broad-reaching health communication interventions is both necessary and possible without sacrificing quality or accuracy of health messaging. Second, distilling messages to their simplest forms and conveying them in ways that creatively sidestep language, cultural and literacy barriers, must be a priority when rapid, global scaling of health communication is needed. Finally, by leaning on best practices from entertainment-education, communication theory and the animation industry, health messages can be made broadly compelling in times when globally deployable interventions are urgently needed.

## OUR WORK AND THE WORDLESS, GLOBALLY SCALABLE COVID-19 PREVENTION ANIMATION

We are health educators and researchers who specialize in the design of broadly accessible health communication interventions. We develop short, small-file health videos that are shareable via smartphones using minimal data. Two principles guide our content development: 1) the use of narratives to engage viewers and minimize reactance/counter-arguing to the health messages [1,6] and 2) the value of entertainment-education for providing a framework that supports longer term message retention and behavior change [4]. In terms of video development, we use a human-centered design approach, keeping the needs and desires of our target audiences at the forefront, while employing a process of rapid iteration and feedback during the development process [7].

## COVID-19 VIDEO INTERVENTION CONSIDERATIONS

The challenge and opportunity during the current COVID-19 pandemic crisis is to create content that can be rapidly deployed globally (so is linguistically and culturally agnostic) while remaining engaging enough to reach a variety of audiences. As a health issue, COVID-19 presents an extra challenge because many in the general public do not view it as potentially affecting their own lives (because the virus is thought to be geographically distant or only likely to affect the medically frail).

**Figure:**
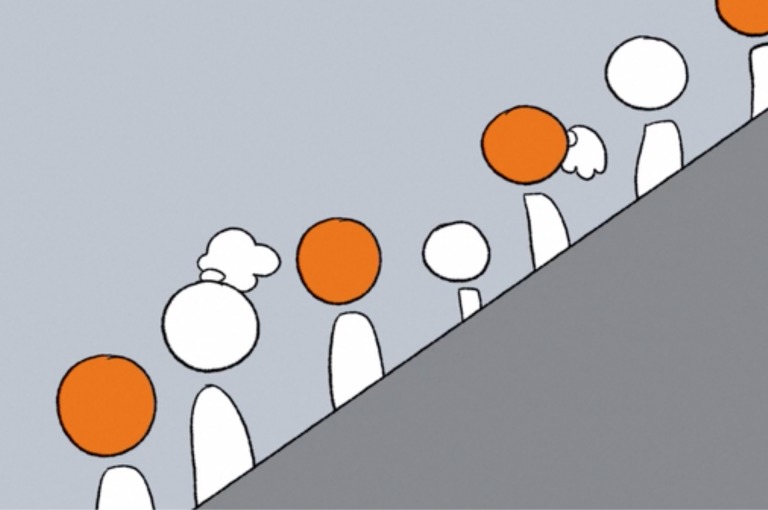
Photo: Scene from a wordless, globally scalable COVID-19 prevention animation. Available on YouTube. Source: ©Stanford Medicine 2020, used with permission.

For our video intervention, we chose key messages based on the WHO recommendations and focusing on (a) how the novel corona virus is spread and (b) what can be done to stop the spread. We focused on “evergreen” messaging – information that was unlikely to change and could thus be impactful throughout the pandemic. These included frequent handwashing, social distancing, avoiding touching your face with your hands, and self-isolating when sick. We also underscored that panic and hoarding (hallmark behavioral responses to the pandemic) were not helpful.

We designed a simple storyline to convey the priority health messages. We gathered rapid feedback on a set of simple, culturally agnostic characters to convey the primary routes of viral spread and the main preventive strategies. When deciding on the tone of the video, we weighed the desire to reassure viewers with the need to underscore the seriousness of the pandemic, especially given media reports of young adults around the world ignoring public health recommendations. The first half of the video uses tense music that emphasized the urgency of the situation; the second half uses uplifting music that is meant to inspire hope and feelings of self-efficacy. Sound effects were strategically used to enhance engagement in the story. We made a concerted decision to keep the video short (less than 2.5 minutes) to sustain attention and facilitate video sharing.

The Stanford Medicine Communications office featured the video first on their YouTube channel. It was later posted on all Stanford social media channels. In the interest of time, we made an early decision not to create tailored videos for different demographics, but rather focused on a single approach that we hoped would resonate globally.

## COVID-19 VIDEO INTERVENTION ITERATIONS AND FINAL PUBLIC RESPONSE

Our video went through two iterations in a 24-hour time span. Each iteration was shared with colleagues in our global health network including a) the communications team at Stanford Medicine b) global health academics, scientists and advocates in Germany, South Africa, the United Kingdom and Canada, and c) community health workers (CHWs) in South Africa. Feedback from CHWs was gathered via WhatsApp and summarized by local supervisors. Feedback from researchers and fellow health educators was solicited through email and text messages.

The first video iteration began in a wet market where the virus made its first jump from a cut of meat onto a person’s hand, before spreading globally. The wet market scene was altered, then removed entirely, in response to concerns that the scene might contribute to xenophobia and the stigmatization of cultural food traditions in East Asia.

In the first and second iterations, several academics felt the first part of the video, which outlines how the virus is spread, was overly anxiety-provoking. Non-academics felt the tension-building helped to sustain engagement and drove home the message that viewers needed to take public health recommendations seriously. We ultimately decided to strike a balance. We retained the storyline but slowed and softened the fast-paced, dramatic music. We ultimately felt that a degree of tension-building was necessary to maintain engagement, especially among those who were defying public health recommendations. Building tension towards a central climax also aligned with best practices for using dramatic storytelling to alter audience behavior [3].

**Figure 1:**
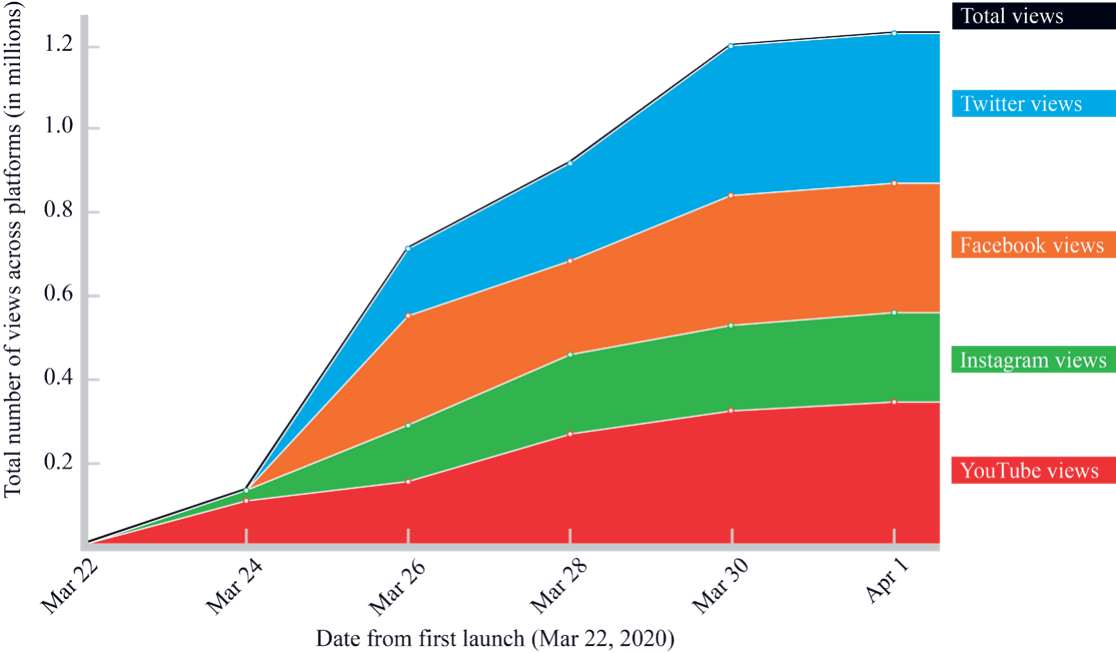
Total number of views by platform across a 10-day period following first release (22 March 2020).

We submitted a final video prototype to Stanford Medicine on 18 March 2020. The communications team was eager to release video content that could make evidence-based health messages accessible to global audiences and had been consulted throughout the intervention process. They therefore expedited the review and approvals process, posting the video on Stanford Medicine’s YouTube channel on 22 March 2020. This was followed by postings on Stanford University’s Twitter, Facebook and Instagram accounts. The video began going viral within 24 hours. Within ten days, it had reached 332K views (YouTube), 220K views (Instagram), 294K views (Facebook) and 402K views (Twitter). Ten days after its release, across only the Stanford social media platforms, the video had reached more than 1.2 million views. (**Figure 1**) Additionally, the lead author, whose contact appears at the end of the video, received email requests to repost the video on external social media channels from 23 private and public sector health agencies, consulates, military organizations and major media outlets, in six regions (Sub-Saharan Africa, North America, South America, Western Europe, Eastern Europe and Southeast Asia). Interestingly, several international members of the Deaf and Hard of Hearing communities also reached out to convey thanks.

## CONCLUSION

In order for global public health messages to be rapidly disseminated, they must be equal parts compelling and accessible to diverse global viewers. Rapidly designing these for the broadest target audience by a) eliminating text, b) using culturally agnostic animations and compelling soundtracks, can yield socially inclusive messages with broad public appeal. By leaning on best-practices from entertainment-education, communication theory and the animation industry, evidence-based public health messages can be presented in broadly compelling ways, meeting the health communication needs of our diverse global community.

**Acknowledgment:** The authors would like to acknowledge our colleagues and collaborators around the world who gave us invaluable feedback on the early video prototypes. Thank you for your generosity in sharing your time and expertise with us on this project.

**Funding:** The video intervention was funded by Stanford Medicine.

**Authorship contributions:** Dr. Adam led the development of the video intervention and co-authored the commentary. Dr. Bärnighausen and Dr. McMahon advised on the intervention development and co-authored the commentary with Dr. Adam.

**Competing** **interests:** The authors completed the ICMJE Unified Competing Interest form (available upon request from the corresponding author), and declare no conflicts of interest.
